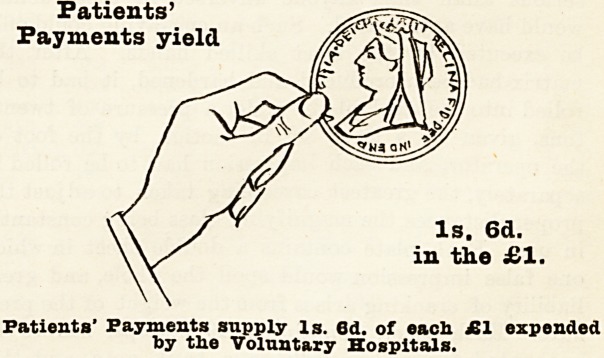# Special Hospital Sunday Supplement

**Published:** 1897-06-19

**Authors:** 


### Special Hospital Sunday Supplement.

### Hospital Sunday and the Jubilee.

Hospital Sunday comes round again at a moment when people's minds are full of other things, and unless a great effort be made to arouse an interest in it and all that it means to Londoners, its coming may be forgotten and its utility may be impaired. Nothing could be more sad, and we feel sure that nothing could be more distressing to Her Majesty, than to find that while the nation is celebrating so great a Jubilee and is uniting in offering respectful congratulations to its beloved Sovereign, the sick in hospitals should be forgotten, and the usefulness of our great medical charities should be lessened.

Cavillers have even asked why Hospital Sunday should have been allowed to fall on such a day. To these it must at once be answered that when a great organisation has at great labour been built up, and when by repetition, year after year, the devotion of a special Sunday to a special work of charity has become part and parcel of London life, to change the date would mean the practical destruction of the Fund. No! It had to be; and we ask, is it not most appropriate that charity should go hand-in-hand with rejoicing, and that at a moment when there is every sign of money being spent freely and with an almost lavish hand, we should step in and remind those who are opening their purse-strings for so many purposes—for festivities, for Jubilee seats, for the entertaining of friends; and, we may add in many cases, for personal decoration and personal enjoyment—that all the time our courts and alleys are full of sick, that accidents are happening every day, that bread-winners are being laid low, that bonny children are being robbed of their bright looks and are being worn down by disease, and that the only help these people can look for, and, in too many cases, the only thing that stands between them and death, is admission to those hospitals for which we now plead, hospitals whose resources and whose powers of doing good are threatened by the prevailing tendency of Londoners to think of only one thing at a time, and that thing in this case festivity. A tithe of what is now being spent in pleasure and in sight-seeing would set the hospitals on their feet and would relieve an untold amount of misery.

What we have to face is that the hospitals are short of money, and that without money they can do nothing; that the hospitals have so grown into the social life of London that the continuance of them is an absolute necessity; and that our voluntary hospital system is so organised that, while its direct benefits go to the relief of accident and sickness among the poor in a manner which would be impossible by any other agency, its indirect benefits are spread over all classes.

No one can stand aloof and say that he at least has nothing to thank the hospitals for. Every one who, either in himself or in the person of his relatives or friends, receives the attention of a doctor or a nurse in sickness, has to thank the hospitals for the training by aid of which they are enabled to ease his pain and cure him of his disorder. Nor can we stop there. So far as hospitals have served as medical schools, they have educated that great band of sanitary experts who devote their lives to the prevention of disease. Even the strongest and healthiest among us is thus personally beholden to our hospitals.

To all this we would add that the hospitals belong to the whole of London; that they are not mere local institutions; and that by gathering together all classes and all districts to unite in one great effort to support these institutions in which the whole of London shares, Hospital Sunday serves a great and useful purpose. In saying this we include, of course, the suburbs.

No greater social change can be imagined than that which is involved in the growth of suburban London. Thousands and thousands of people every night leave the business parts of town for suburbs where tens of thousands of people dwell who, for their daily food and clothing, for their houses and their gardens, and for every pleasure that they have, are dependent upon those who do daily work in this great central mill which we call London. Nor do these suburbans now live in exile as they used to do. The shops, the picture galleries, the exhibitions, the theatres, and the concerts are all crowded every day with suburban people, who by dint of railways are now as truly Londoners as those who dwell within the circle and breathe the smoke of town. If we look in at any suburban railway station late in the afternoon we see trains filled with suburban people going to take their evening's pleasure in London; if we look in the morning, these trains are filled by men going to earn money in London; if we look in the middle of the day, we find the sick and the cripples going up for help and assistance from the London hospitals; in every sense the suburban dwellers share the advantages of London, and we hope that they will see how strongly bound they are to support its charities, and especially the Hospital Sunday Fund.

Again, we repeat that, although it will require energy, hard work, and careful organisation to prevent Hospital Sunday this year being a failure, in consequence or the many distractions before the public mind, yet it is intensely appropriate, not only that it should fall at this particular season, but that it should be made a grand success. We can think of nothing more fitting, and nothing which could add greater lustre to the Jubilee celebration which will take place almost as the collecting-bag is going round, than that in the midst of their loyal rejoicing people should give of their substance to the poor, and should offer substantial help to the hospitals of London which exist for the benefit of those for whom Her Majesty is known to feel such sympathy—the sick, the suffering, and the afflicted.

### THE DISEASES FROM WHICH LONDONERS SUFFER.

IN order to show the proportion borne to one another by the various classes of disease from which the inhabitants of London suffer, we have prepared the following two sets of diagrams, which are all drawn to scale, so that the size of the several squares in which the little drawings are contained are proportionate to the number of patients applying to the hospitals in the years 1894 and 1895 for the treatment of the different classes of disease which are named below.

The cases which we have sorted out in this way comprise those treated at the voluntary hospitals and dispensaries of London—including the endowed hospitals, St. Bartholomew's, Guy's, and St. Thomas's—and also at the hospitals of the Metropolitan Asylums Board. They amount altogether to the enormous total of *one million seven hundred and fifty-three thousand six hundred and eleven patients.* All these were treated during one year, viz., 1895, and to this year only the letterpress refers, although the figures and proportions for both 1894 and 1895 are shown in the diagrams.

**Diagrams of Diseases.—1894.**

**810,703 Surgical Patients.**

**622,816 Medical Patients.**

**124,209 Children.**

**109,407 Eye.**

76,125 Women.

46,522 Consumption.

43,472 Throat & Ear.

48,902 Skin.

19,224 Fever.

13,664 Paralysis.

**Patients Suffering from Surgical Diseases.**—Of the whole number of patients received by the hospitals *eight hundred and forty-seven thousand four hundred and seventy-six* required surgical treatment. What is meant by "surgical diseases"? They include all accidents, *i.e.*, broken bones, smashed limbs, fractured skulls, and all manner of fractures, displacements, and crushings of sensitive parts or organs. They further include abscesses, ulcerations, cancers, and tumours of all kinds; and, indeed, every injury which accident or pathological process may produce. Surgical diseases include all accidents and all lesions which may be dealt with by hand or instrument. Let anyone who desires to realise what surgical diseases mean for the population of London try to realise this army of 847,476 persons suffering from one or more of the injuries here briefly summarised.

**Patients Suffering from Medical Diseases.**—*Five hundred and fifty-eight thousand three hundred and nine* cases received medical treatment. What does the medical profession mean by medical diseases? Diseases which are situated in their entirety or as to their source and origin in one or other of the three great cavities of the body, many of them deep-seated, most of them removed from sight, the diagnosis of their nature and extent is dependent upon the scientific knowledge of the doctor to whose treatment they are committed. They include, rheumatic fever, pneumonia, pleurisy, bronchitis, every kind of heart disease, many forms of brain lesion, diseases of the stomach, bowels, liver, kidney, bladder, pancreas and spleen, most nervous diseases, dyspepsia, constipation, headaches, sleeplessness, and a myriad other ailments, many of them serious, and often resulting in grave danger to life, or at least to the useful existence of mankind. Imagine for a moment considerably more than half a million human being in London alone attended, free of cost to the patients, by the leading physicians of the day, within the buildings of the hospitals of London.

**Patients Treated at Special Hospitals for Children.**—*One hundred and twenty-seven thousand two hundred and seventy-two* young people greviously ill sent from homes where they could not be properly treated or even carefully attended to. The illustration which we issue with this Supplement will bring to the mind of every sympathetic person who studies what children have to suffer, how excellent is the skilled medical and surgical care which the hospitals supply to the many cases which arise amongst a large population like that of the metropolis of the British Empire. Surely no words are needed to make the younger residents in this great city determine to give something, even from a limited income, to help suffering children to secure a return to health, and the power of one day shielding themselves against the dangers and risks to life of a vast city like London.

**Patients Suffering from Eye Affections.**—*One hundred and nine thousand and twenty-eight* persons were treated in the special departments of the general hospitals or by the ophthalmic hospitals of London. It will be seen that, apart from the blind, there were over one hundred thousand people suffering from various forms of disease of the eye which often entailed excruciating pain, and must in many cases have terminated in loss of sight had it not been for the treatment they received at the hospitals. Those who are blessed with sight will surely give something to the hospitals on Hospital Sunday as a thank-offering for their escape from one of the most cruel diseases to which the human frame is liable.

### Diseases from which Londoners Suffer—*continued.*

**Diseases of Women in Motherhood.**—*Seventy thousand six hundred and forty-nine* women were treated at the metropolitan voluntary hospitals, the major portion of whom attended the special hospitals for women and the lying-in institutions. Sons of good mothers, and, indeed, all sons must necessarily be moved to pity by realising that, apart altogether from the diseases to which all are liable, woman has to face others which are peculiar to her sex and which entail an immensity of suffering, if they do not render the sufferer permanently disabled from the enjoyment of life and the pursuit of occupations which render life happy and profitable. This group of diseases must excite the sympathies of the most careless, and we make bold to believe that it will stir up many persons to give something this year to the hospitals if they have never yet enjoyed the luxury of investing a handsome sum in so noble a cause.

**Patients Suffering from Consumption.**—*Forty-seven thousand two hundred and thirty-six* persons suffering from phthisis or consumption were treated at the consumption hospitals of London during the year. Consumption is the curse of our climate. It spares neither the peer nor the pauper. It attacks the young, the old, and the middle-aged, whilst its ravages defy the highest skill of the greatest physicians of modern times. Who can say with certainty that he may not fall a victim to this dire disease, and who can find it in his heart to deny something towards the cost of alleviating the sufferings of those who have been stricken with this terrible malady?

**Patients Suffering from Diseases of the Ear and Throat.**—*Forty-three thousand two hundred and ninety* persons were treated at the special hospitals or special departments devoted to these diseases. The ear, throat, and nose are intimately connected, and large numbers of people require a visit to one of the special hospitals devoted to the ailments included in this section. Residents in a city like London, including both children and adults, are specially liable to these affections, which may involve temporary or permanent impairment of hearing, swallowing, breathing, and even speaking. Consider what it would mean to anyone of us to suffer from one or all of these affections. A little quiet reflection will surely awaken sympathy for those who thus suffer.

**Patients Suffering from Diseases of the Skin.**—*Thirty-nine thousand one hundred and thirty-five* patients were treated for skin diseases in London during the year. When it is remembered that many people of both sexes are terrified by the appearance of any affection of the skin, even in these days of advanced science, when immediate alleviation is possible, and permanent relief in the majority of cases assured, little need be said to awaken the sympathy of the reader for the many thousands who annually suffer from ailments of this description. We confidently claim a liberal offering from every inhabitant of London towards the funds needed to rescue those who have to suffer from the extremely common but essentially disagreeable illnesses included in this section.

**Patients Suffering from Fever.**—The number of people who suffered from the various forms of fever during the year amounted to 21,811. Of this number by far the greater proportion were treated at the hospitals of the Metropolitan Asylums Board. But the term fever includes much besides the class of fevers which are usually removed to these hospitals. All over London measles prevails so largely as to cause three times as many deaths as occur from scarlet fever, and more even than take place from diphtheria, and all these innumerable cases of measles have to be attended in the homes of the people. Moreover, of the diseases notified in 1895 43 per cent. of the scarlet fever cases, 66·3 per cent. of the diphtheria cases, and 81·2 per cent. of the typhoid fever cases were not removed to the Asylums Board hospitals. Except a few of the typhoid cases which were taken to the general hospitals, and some of the diphtheria cases which were carried off to the general hospitals in order that operations should be done to prevent impending suffocation by the disease, the great mass of these patients were treated in their own homes. The treatment of fever in its various forms has then fallen in a very much larger degree than many people imagine to the medical officers of the many charitable dispensaries scattered over London.

**Patients Suffering from Paralysis and Diseases of the Nerves.**—*Sixteen thousand six hundred and seventy-seven* people were stricken with paralysis and diseases of the nerves, and received treatment at the several hospitals devoted to these maladies. In the morning a man rises from his couch in robust health, and leaves his home to pursue his ordinary avocations. Whilst he is in the counting-house, or the study, or the office, or in the shop following his ordinary business, paralysis seizes him, and he is carried home insensible, helpless, and incapable of uttering a word. No disease is more appalling in its suddenness than paralysis, and it should be matter for thankfulness to Londoners that the hospitals and dispensaries of London were able during the year to give succour to no less than 16,677 cases of paralysis and diseases of the nerves. No one can doubt the association of nervous breakdown with the toil and moil of London life. Let those who are still spared give freely to relieve those who have fallen out of the race, and to keep open the hospitals where immediate treatment can be given to whomsoever may be struck down by those diseases.

**Diagrams of Diseases.—1895.**

847,476 Surgical Patients.

558,309 Medical Patients.

127,272 Children.

109,028 Eye.

70,649 Women.

47,236 Consumption.

43,290 Throat & Ear.

39,135 Skin.

21,811 Fever.

16,677 Paralysis.

### A Quarter of a Century of Hospital Sundays.

Standing out prominently among the events of the year 1897 is the Diamond Jubilee of Her Majesty's reign, a reign during which many changes have taken place, but none, probably, of greater moment than the alterations which have occurred in the modes and methods of charity. We still find the wealthy giving out of their abundance to their less fortunate neighbours—never, perhaps, more so—and we see on every side illustrations of the facts that not only is the possession of wealth admitted to involve great duties and responsibilities, but that the wealthy have found out by experience that one of the great pleasures which riches alone can give is the power of so bestowing them as to do good to others, and to earn for the givers the gratitude from the poor and an honourable position among their fellow-men. Never, perhaps, before was it considered so honourable as it as at present to be mixed up with and concerned in works of charity.

The great change, however, which a comparison of the present with the past brings out is the downward permeation of the tendency to give, the introduction of new forms of giving, and the invention of new machinery for the collection of gifts. Among the latter we must place in the forefront the institution of Hospital Sunday; and the intimate relation of this particular Hospital Sunday with Her Majesty's Jubilee, not merely from the fact that it coincides with it in date, but that the memorials by which the Jubilee is to be permanently celebrated are so largely connected with the intervention of Royalty on behalf of Charity, leads us on this occasion to look backwards, and to compare the present with the past.

It is not here a matter of going back to the beginning of the Queen's reign, for Hospital Sunday is hardly a quarter of a century old. We need only go back to 1873 for the first Report of the Council of the Hospital Sunday Fund, and it is easy to see what changes have taken place since that time, and to recognise how great an influence for good the institution of this Fund has been, even outside and beyond its efficiency as a means of collecting money.

Let us first compare the work done then and now. In 1873 the voluntary hospitals and medical charities in London which participated in the Hospital Sunday Fund possessed 5,162 beds; in 1896 these beds had increased to 8,800.

In 1873 the number of in-patients was 41,859, and the number of beds constantly occupied was 4,008, while in 1896 the figures were 88,554 and 6,699 respectively. We see, then, how largely the work of the hospitals has increased during that period. Figures, however, are dull reading, and at best are but an imperfect means of expressing facts. What, then, does this great increase in the work of our hospitals mean? Does it mean that zeal has outrun discretion, and that too much hospital accommodation is being provided? Not at all. Look at the growth of London, and compare its population a quarter of a century ago with what it is now. In 1871 it was 3,254,260, while in 1896 it had risen to 4,392,346, an increase of upwards of a million souls, as the saying is. But these souls are possessed by upwards of a million bodies, a large proportion of which are sick and afflicted, subject to accident, and so lodged in crowded dwellings that they cannot obtain proper medical treatment except within the walls of the hospitals. The figures we have given show, then, that London is gradually increasing its hospital accommodation, and is making a steady effort to provide for its sick and deserving poor without driving them to the workhouse; but they also show that, all the while, the more we do in that direction the more is wanted. While London grows the Hospital Sunday Fund ought to grow, and especially ought it to spread out its tentacles into the suburbs and draw from them an increasing mass of subscriptions.

Let us return to our figures. It will be noticed that while the number of beds constantly occupied has increased by just about one-half (viz., from 4,008 to 6,699), the number of patients treated in them has increased more than cent. per cent. (viz., from 41,859 to 88,554). Evidently the stay of each patient in hospital is now much shorter than it used to be. Now this means two things, both of which should be borne in mind. First of all, it means improved treatment. The period we are dealing now with —*i.e.*, between 1873 and 1896—has witnessed the introduction of those methods of treating wounds which are commonly spoken of as the antiseptic system, and there can be little doubt that it is to this that is largely due the much greater rapidity with which hospital patients are now often cured, as compared with what took place in times gone by. There is, however, we fear, another cause of the shortened stay of patients in hospitals, a cause which the subscribers to the Fund should bear well in mind, viz., the constant pressure on the hospitals, the crowd of applicants anxious to get in, the urgent necessity placed upon the managers to make the most of their wards by turning the patients out as soon as ever that can be done without danger, and by making a strict selection on admission of cases whose treatment will not take long. This is an urgent, and sometimes what may seem a cruel necessity, and it is one which greater subscriptions can alone prevent. On the other hand, it is to be remembered that there has of recent years been a great increase in the number of convalescent homes, and that one of the best ways in which the pressure on the London hospitals can be relieved is by making it possible to pass their patients quickly on to places where they can have pure air in addition to other advantages.

Perhaps the most striking figures in the tables are those giving the income and expenditure, and from them we learn the necessity for constant giving if the hospitals are to be maintained in full efficiency. The expenditure has more than doubled since 1873 (from £351,109 to £731,790), while the total income has far less than doubled (from £423,234 to £713,538), so that, although when the Hospital Sunday Fund was founded the total income was £72,125 in excess of the total expenditure. it is now £18,262 short. In 1873 the Hospital Sunday Fund was founded in pure charity, to provide for the many that opportunity of sharing in the good work of helping the hospitals which had before fallen to the few. It is otherwise now. Then the hospitals were able to put by something year by year to their reserve funds; now they are spending all they have, and more. Yet no one can go into the homes of the London poor without feeling how absolutely and urgently necessary the hospitals are, and we hope that, bearing these facts in mind, givers of all classes will this year of all years give to the utmost, so that the hospitals may continue in their good work.

### Then and Now.

Accompanying this Supplement of The Hospital, we present to our readers a full-page illustration which depicts a ward in the early days of Her Majesty's reign, and one which is to be seen by paying a visit to a complete and modern hospital in 1897. Comparison shows very strikingly the sentiment, if we may so call it, which has gradually converted the severe barrack-like apartment into a delightful haven of rest for sufferers, almost palatial in general appearance, yet homely in the comfort it affords. Glancing at the representation of the earlier ward, we cannot be altogether surprised that a feeling of dread and despondency should have been experienced on entering a hospital. Some decades ago, clean and wholesome and spacious as the ward might be, it yet was so devoid of the decorative element, so comfortless for the semi-convalescent, that there was nothing to reconcile the sufferer to his surroundings, or distract his thoughts during weary hours of pain. During the reign of Victoria all this has been changed. Hospital managers, the nurses, and the public, all now lend a helping hand to beautify the abodes of suffering, and to hasten cure by the aid of bright surroundings with all their pleasant suggestiveness. Who shall say that the womanly influence of our Queen, with her tender solicitude for the afflicted, has not influenced the sentiment of her subjects, and shown that to all is given the power to participate in the great work of healing? No Royal family has ever presented such an example to a nation in acts of sympathy and self-sacrifice in the cause of suffering humanity as ours. The perusal of the newspapers bears daily witness to this, and with such encouragement no one should stand aside or refuse to share in this good work.

Then, again, the Queen's reign provides no more startling contrast than that between the old nurse and the new. The latter does not owe her existence to past conditions. She is a direct product of the age. Between her and her Gamp-like predecessor there is no link. She came suddenly as an invader, and conquered —not without a battle here and there! Our illustrations show the trained nurses as they were in the middle of the present age, and the modern nurse, as she is to be met now, in the wards of the hospitals of to-day. Young and neat as was the nurse in the earlier days of training, there was an absence of the professional smartness characteristic of the most up-to-date training schools of to-day. The condition of things existing previous to her advent is difficult to realise. It is not too much to say that those engaged in nursing were the very dregs of womankind. Confirmed drunkenness was the only disqualification, the question of respectability being ignored. Indeed, the calling was recognised as disreputable and unfit for self-respecting women. In such circumstances it is not surprising to read that "every vice was rampant" amongst the nurses. It is not difficult to imagine how patients fared in their hands. There is a gruesome tradition of the bed-clothes being removed to hasten the end of the dying. At night they might at least depart in peace, for then the nurses were relieved by persons called with grim but unconscious humour, "night watchers," who dozed in an armchair, or made themselves comfortable on a patient's bed. Little wonder that the poor had a horror of hospitals. All kinds of cases, medical and surgical, were herded together, and the nurses devoted to them such time as they could spare from their scrubbing and cooking. Much of the latter was done on the ward fire, adding another savour to the burdened air. Vermin was prolific. Several causes combined to maintain this appalling state of things. Foremost was the fact that in Protestant England religious nursing was almost unknown. Strange as it may seem at the present day, the nursing work performed by sisterhoods on the Continent was in advance of anything to be seen in England. Again, the conditions were such that, in the absence of some higher motive than mere gain, hospital nursing was repellent to any but the coarsest natures. Hygiene was in its infancy; antisepticism was unknown; and, above all, there was no chloroform. What an operation meant in those days is vividly pictured by Mr. Treves in the current number of *The Practitioner*. To those interested in the progress of hospital work we cordially recommend a perusal of the whole article. "Sixty years ago," writes Mr. Treves, "the operating-theatre was the dirtiest room in the hospital. There was no apparent reason why it should be otherwise." And the patient! He "received scant attention as he lay upon the table with his eyes turned to the rafters, listening acutely for that footstep which would tell him that the hideous moment had come." Callous women were required to face such horrors. Consequently the introduction of chloroform has indirectly played an important part in the evolution of the trained nurse. Her genesis is not difficult to trace. In 1840 Mrs. Fry and Lady Inglis had founded a nurses' institute for paid sisters—the first of its kind in London. From this time onwards religious sisterhoods played an important part in the furthering of nursing work. St. John's House was opened in 1847. In the following year Middlesex Hospital provided accommodation for resident superior

Trained Nurses of the Mid-Victorian Era.

A Trained Nurse of To-day.

nurses—"the inferior nurses apparently still coming in, with the milk, in the morning, as the charwoman does now." Other institutions, notably King's College Hospital, were working in the right direction. The change had set in, but public interest in the movement was wanting. Under such conditions, many years might have passed without definite reform. An opportunity, however, offered in 1854 for rousing the enthusiasm of the nation. Humanity owes it to Miss Florence Nightingale that the opportunity was seized. She not only possessed all the natural qualities required for her great task, but had fitted herself for it by years of unselfish effort. The story of her work is too well known to require retelling. She has earned for herself a place in the history of the century. The enthusiasm of which she was the centre gradually spread among the women of England. Nursing gained recognition as a good work, and was taken up in a spirit of self-sacrifice by those who would, a few years before, have shrunk from it as menial or worse. The grosser features of hospital life were by this time modified, and an added refinement soon resulted from the presence of the new comers. In 1860 the Nightingale Training School was opened in connexion with St. Thomas's Hospital. As time passed the field widened. In 1859 the first district nurse began work in Liverpool, and six years later Miss Agnes Jones, with twelve nurses from St. Thomas's, introduced the new methods into the workhouse infirmary of that city. In 1869 Mrs. Deeble became the first superintendent of Her Majesty's Nursing Sisters, and started work at Netley with six Nightingale probationers. Henceforward development in every direction was marvellously rapid. At the outset the new type of nurse regarded her work as purely philanthropic, but soon it became evident that educated women were converting it into a calling as admirable as in the old days it was horrible. During this period both medicine and surgery have undergone considerable changes. Medical treatment has become much more complex, the management of cases being recognised as of greater importance than the administration of drugs. This has thrown an increasing amount of semi-scientific work on the nurse, and it has been found necessary to impart to her special knowledge sufficient to enable her to follow intelligently the physician's methods and to understand something of the reasons underlying the instructions she has to carry out. On the surgical side the introduction of antisepticism and asepticism has evolved further responsibilities, to meet which the nurse requires careful theoretical and practical training.

Such, briefly, is the history of the modern nurse. Champions of the "untrained angel" may decry her, but those who know the brave heart with which she faces her arduous duties, her unwearying if quiet sympathy, and her unconscious self-sacrifice, can form a truer estimate of her worth. She is seen at her best in the hospitals, for which she has done so much—how changed from the lazar-houses of old! Where dirt and confusion abounded everthing is now spotlessly clean and well ordered. With the description of the old operating-room in our minds, we are tempted to visit the new one. The walls are tiled; the tables are made of glass. There is no corner where a speck of dirt can lurk undetected. The operator is clothed in white—the patient feels nothing. Could the contrast be greater? In the wards the signs of suffering are few. The great chambers, with their double row of beds—no longer crowded together so that they almost touch—are a picture of comfort. There are engravings on the walls, and plants and flowers on the tables; often, too, a piano may be seen. Quiet and contentment reign everywhere. Truly things have changed, and for the better! J. B. AND M. D.

### A WORD TO LIVING LONDONERS.

### WAYS AND MEANS.

IN previous years we have pointed out that if the 1s. 6d. in the pound derived from patients' payments be credited to the living, *i.e.*, to the present inhabitants of London, then the sum yielded to the hospitals from the living has only just about equalled that contributed by deceased benefactors, and we have urged the press to spread these facts widely, so that the living might arouse themselves and increase their contributions on Hospital Sunday so as to make the living hand give at least eleven shillings out of every sovereign expended upon the care of the sick to-day. We rejoice to notice that the returns for the year 1895 show that, including St. Bartholomew's and St. Thomas's Hospitals, the living gave 8s. 9d. in the pound, or 4d. more than in the previous year; and, if we add to this sum the 1s. 6d. in the pound derived from patients' payments, it brings the contributions from the living up to 10s. 3d. in the pound. This result is encouraging, especially when taken in conjunction with the fact that there has been a steady increase in the amounts derived from the living during the three years 1893, 1894, and 1895, and that in 1896 the contributions of the living equalled, if they did not exceed, the amount given in 1895. But—and there is always a "but" in matters of this sort—as we point out more fully further on, we must not allow this encouragement to render the exertions on behalf of the medical institutions any the less vigorous at the present time.

### THE COST OF THE WORK DONE.

In the year 1895 the total expenditure of the London voluntary hospitals (including St. Bartholomew's, Guy's, and St. Thomas's) and the metropolitan dispensaries was £808,926. In other words, it required upwards of four-fifths of a million of money to defray the cost of providing adequate hospital treatment for the patients, who numbered about one million and three-quarters (1,736,000). We have excluded from this calculation 19,360 fever cases treated at the hospitals of the Metropolitan Asylums Board, and the cost of such treatment.

The total income of the London voluntary hospitals and dispensaries during 1895 was £852,400, which was derived from the following sources:—

| | |
|---|---|
| Charitable or voluntary contributions ... | £353,154, or 42 p.c. |
| Income from invested property ... ... | 257,141, or 30 p.c. |
| Legacies ... ... ... ... ... | 181,225, or 21 p.c. |
| Patients' payments... ... ... ... | 60,900, or 7 p.c. |

In the above figures the income and expenditure of St. Bartholomew's and St. Thomas's Hospitals, being £122,544 and £108,795 respectively, have been confined to that portion of their revenue and expenditure which was applicable to hospital purposes.

### HOW THE MONEY IS PROVIDED.

Let us now consider where the money came from to pay the cost of the hospital relief given to the inhabitants of London by these voluntary institutions. In order to bring home the facts to the meanest compre-

### "THEN AND NOW."

[AN EARLY VICTORIAN WARD.

*(Reproduced by the kind permission of the Treasurer of St. Thomas's Hospital.)*

A WARD IN 1897.

hension, we have prepared diagrams each representing a hand and a coin, which have been drawn to scale, and which show exactly the proportion of every sovereign expended which was contributed on the one hand by the living, who received all the benefits, and on the other by deceased benefactors, many of whom took an active part in the management of hospitals during their lifetime, and whose benefactions have enabled these institutions to meet the ever-increasing needs of upwards of four millions of people. With a view to clearness and ready comprehension, the diagrams have been drawn so as to represent the proportion given of every sovereign expended in 1895 by (*a*) the living, (*b*) the dead, and (*c*) the patients themselves. *The black hand* and the coin held by it represent the dead hand, *i.e.*, the contributions from those now dead, *the white hand* represents the charitable contributions of the living, and the smallest coin the amount derived from patients' payments.

**The Dead Hand gave**

**10s. 10d. in the £1.**

**The Dead Hand gives 10s. 10d. out of every £1 expended by the Hospitals.**

Of every sovereign expended **10s 10d.** or rather over *one-half, is derived from legacies and the interest upon gifts* of deceased benefactors which have been invested in approved securities; **8s 9d.** out of every sovereign expended has been *given in charity by* the present inhabitants of London, that is, *the living, for whose benefit the hospitals exist*; and **1s 6d** out of every sovereign has been *contributed by the patients treated in the hospitals.* From this it will be seen that in the year 1895, with which we are now dealing, hospitals, as a whole, received 21s. 1d. for every 20s. they spent. It must not be imagined, however, that this implies that the living have now reached the maximum amount that they need contribute, or that they can in any way relax their efforts on behalf of the hospitals. We have only to look carefully at the figures and consider what they mean to realise this at once, for it will be seen that the greater portion of this surplus (10d., in fact, out of the 1s. 1d.) is due to the large increase in legacies—always a very fluctuating source of income and one not to be depended on in any way. A striking illustration of the fluctuating nature of receipts from legacies is given us if we glance at the figures for 1896, when we find that the hospitals and dispensaries of London received about £40,000 less from legacies in that year than they did in the previous year (1895), with which we have been dealing. This falling off is equivalent to about 1s. 2d. out of every sovereign expended in 1896, so that the surplus which we might have been inclined to congratulate ourselves on in 1895 more than disappears, and we have to fall back at once on the other sources of income, not for a surplus, but to make up the bare amount necessary to meet the expenditure. We therefore arrive at the result that the increase in 1895 on which we can rely (*i.e.* the increased receipts from subscriptions and charitable contributions and patients' payments) is only 3d. in every sovereign expended, an increase which, while it leads us to be hopeful (especially as it is sustained in 1896), is not an increase which—having regard to the ever-growing demand upon the hospitals and the rapid extension of the population of London—is sufficient to make us say that the inhabitants of the Metropolis have done their duty by those institutions which care for the sick poor.

**The Living gave**

**8s. 9d. in the £1.**

**The Living, i.e., the present inhabitants, only give 8s. 9d. of each £1 expended by the Voluntary Hospitals.**

THE MEANING OF THE DIAGRAMS.

We desire to direct the attention of all classes in London to the foregoing figures. They show that, whereas the people of London resort to the voluntary hospitals in greater numbers than the population of any other city in the United Kingdom, they still fail to recognise their full duty to the hospitals and to show their sense of the benefits they have received from these institutions. Every provincial city of

**Patients' Payments yield**

**1s. 6d. in the £1.**

**Patients' Payments supply 1s. 6d. of each £1 expended by the Voluntary Hospitals.**

importance takes the deepest pride in its hospitals and provides them with adequate funds. It follows that in provincial cities, instead of the hospitals being starved and impoverished for want of liberal support at the hands of the living, who are continually demanding and always receiving increasing benefits from these institutions, all classes of the population combine to provide the necessary funds in adequate proportions. In London almost the exact opposite is the case. The living show their appreciation of the hospitals by demanding greater value in relief every year at the hands of the hospitals, whilst they contribute in payment for the benefits they receive about one-half of its total cost. Is there one intelligent citizen of London who will be content to find that the population of this vast city contributed so small a proportion of the sum which was expended in affording hospital relief to the citizens as a whole?

Had it not been for the contributions of the dead hand, more than half the hospitals must have been closed for want of funds. We appeal to the people of London to exhibit more self-respect and a determination to henceforth follow the noble example set them in this matter by their forbears and sires.

We hope that the Press will make these facts widely known, and that the living will arouse themselves sufficiently and so increase their contributions on Hospital Sunday this year that the charitable contributions, combined with patients' payments, will at least amount to 11s. in every sovereign. Although it is true that the London hospitals as a whole received in 1895 more than they expended, still in 1896 the income as a whole only about covered the expenditure, whilst there are hospitals like Guy's, Charing Cross, and King's College which are urgently in need of large sums to defray current expenses, and the district of South-east London, containing 1½ millions of the population, has no adequate hospital accommodation at all, a fact which has been making itself felt with increasing urgency for some time past.

### THE PRINCE OF WALES'S HOSPITAL FUND STAMPS.

ONE of the new modes of raising money for the hospitals which have marked the progress of the Prince of Wales's Hospital Fund has been the issue of the Prince of Wales's Hospital Fund Stamps. These stamps, issued for the benefit of this fund, are now on sale to the public, and can be bought at all booksellers, stationers, and newsagents, with the exception of the railway bookstalls. They are issued to give small subscribers a handy and convenient form of receipt, and one which they can retain as a memento of the Diamond Jubilee, and of that increasing interest in hospital support and management shown during her Majesty's reign, not only by the Royal family, but by all classes throughout the country, and culminating in this effort to set the hospitals of London on a sound financial basis. It is also evident that stamp collectors are largely interested, and spaces for these stamps are being made in many new albums, in addition to the issue of specially-prepared pages for insertion in existing albums. The basis of the design selected by the Prince of Wales is taken from no less an authority in art than Sir Joshua Reynolds, whose well-known picture of "Charity," executed for one of the Virtues in New College Chapel, Oxford, is the most appropriate design that could have been selected, embodying as it does a beautiful picture, with Mrs. Sheridan as the chief figure.

After His Royal Highness had approved of the design the engraving was begun, and proved a very much more serious affair than anyone unversed in these details would have anticipated. Such an engraving could only be executed by the most skilled hands. After the matrix had been produced and hardened, it had to be rolled into the steel plate, under a pressure of twenty tons, given by a lever set in motion by the foot of the operator, and each impression had to be rolled in separately, the greatest care being taken to adjust the proper distances, the magnifying glass being constantly in use. Each plate contains a double sheet in which one false impression would spoil the whole, and great liability of cracking arises from the weight of the pressure. Each sheet, containing 40 stamps, has to be accounted for as carefully as a bank note, and this again entails still greater surveillance.

It is needless to say that the fact of H.R.H. the Duke of York being President of the Philatelic Society insures the greatest interest being taken in these stamps by collectors in all portions of the globe, and as a work of art there is nothing wanting in them.

To Mr. Burdett is due the success of the scheme, while valuable assistance has been rendered by Mr. de la Rue and by Mr. Purcell, C.B., the Controller of Stamps at Somerset House, who have taken the greatest interest in the undertaking from its commencement.

It will be remembered that in the case of the Rowland Hill post-card in 1890, so great was the demand that the value of the post card advanced no less than 2,500 per cent., and though the trade is supplied by Simpkin, Marshall, Hamilton, Kent, and Co. (Limited), Stationers' Hall Court, London, E.C., the stamps can be bought at all booksellers', stationers', and newsagents', with the exception of railway bookstalls.

### WHY THE STAMPS WERE ISSUED.

In THE HOSPITAL for the 5th inst., page 166, the history of the stamps was given. It may be interesting to indicate why they were issued. The Prince of Wales from the outset was most anxious to make it readily possible for everybody who wished to be associated with the form of commemoration for Londoners approved by the Queen to give a subscription of from one to ten shillings annually to the hospitals. The objections attaching to street and house-to-house collections under local committees have been so conclusively proved by the working of the Hospital Saturday Fund and the Lifeboat Saturday movement, that this plan was necessarily negatived. There remained the plan of organizing the payment of these small subscriptions through the Post Office, the vestry, the School Board, water-rate, gas, or County Council collectors, with the consent of the authorities if this could be obtained. After full inquiry, each of these sources was found open to objection, so the plan of issuing sufficient stamps to raise a net annual sum of £50,000 was adopted. It has very great advantages, is simple, effective, and free from objection, whilst it secures that all the money given shall be promptly placed to the credit of the Fund. We can only hope that the working classes may be alive in time to the importance of securing a shilling stamp at once, or they may have to pay a premium to enable them to possess one.

### Metropolitan Hospital Sunday Fund, 1897.

### THE WORK OF THE HOSPITALS AND MEDICAL CHARITIES OF LONDON.

### NEWINGTON AND SOUTH DISTRICT.

Comprising Battersea, Wandsworth, Tooting, Balham, Streatham, Brixton, Lambeth, Newington, Southwark, Bermondsey, Camberwell, Greenwich, Deptford, Lewisham, Blackheath, Woolwich, &c.

| No. of Beds. | No. of Beds Daily Occupied. | HOSPITALS. | 1871-73. In-patnts. | 1871-73. Out-patients. | 1894-96. In-patnts. | 1894-96. Out-patients. | Total Expenditure. | Income. Charitable. | Income. Proprietary. | Income. Patnts' Pymnts | Total Income. |
|---|---|---|---|---|---|---|---|---|---|---|---|
| | | | | | | | £ | £ | £ | £ | £ |
| 621 | 420 | Guy's ... ... ... ... ... | ... | ... | 6,249 | 71,730 | 44,006 | 19,634 | 24,309 | 4,776 | 48,719 |
| 25 | 21 | Miller and Royal Kent Dispensary ... | ... | 5,919 | 305 | 16,239 | 3,511 | 2,812 | 272 | ... | 3,184 |
| 253 | 199 | Seamen's... ... ... ... .. | 2,051 | 1,552 | 2,616 | 16,906 | 16,317 | 10,963 | 3,886 | 133 | 14,982 |
| 66 | 40 | Evelina, for Children ... ... ... | 272 | 20,895 | 567 | 6,256 | 4,808 | 6,598 | 655 | 54 | 7,307 |
| 57 | 51 | Home for Sick Children ... ... | ... | ... | 244 | 1,790 | 2,769 | 1,072 | 202 | 360 | 1,634 |
| 24 | 19 | General Lying-in ... ... ... | 307 | 860 | 508 | 1,833 | 3,313 | 728 | 2,516 | ... | 3,244 |
| 36 | 14 | Clapham Maternity and Dispensary... | ... | ... | 312 | 5,838 | 2,231 | 444 | 828 | 838 | 2,110 |
| 52 | 44 | Royal, for Children and Women ... | 131 | 4,793 | 528 | 7,811 | 4,027 | 2,104 | 786 | 261 | 3,151 |
| 42 | 24 | Royal Eye ... ... ... ... | 91 | 3,524 | 426 | 16,228 | 3,615 | 2,530 | 60 | 566 | 3,156 |
| 10 | 4 | Hospital for Diseases of the Skin ... | 15 | 4,160 | 44 | 4,258 | 1,198 | 204 | 171 | 588 | 963 |
| 382 | 196 | Metropolitan Convalescent ... ... | 2,300 | ... | 2,989 | ... | 6,623 | 5,633 | 396 | 115 | 6,144 |
| 10 | 5 | Phillips' Memorial Homœopathic ... | ... | ... | 83 | 3,384 | 577 | 347 | 23 | 196 | 566 |
| 8 | 4 | Eltham Cottage... ... ... ... | ... | ... | 67 | ... | 372 | 337 | 11 | 66 | 414 |
| 20 | 8 | Beckenham Cottage ... ... ... | ... | ... | 150 | ... | 693 | 563 | 43 | 115 | 721 |
| 20 | 11 | Blackheath Cottage ... ... ... | ... | ... | 141 | 880 | 871 | 644 | 20 | 118 | 782 |
| 32 | 17 | Bromley Cottage ... ... ... | 38 | ... | 286 | ... | 1,117 | 744 | 98 | 146 | 988 |
| 14 | 8 | Chislehurst, &c., Cottage ... ... | ... | ... | 113 | 11 | 536 | 391 | 16 | 133 | 540 |
| 12 | 6 | Sidcup Cottage... ... ... ... | ... | ... | 98 | 42 | 434 | 293 | ... | 79 | 372 |
| 7 | 6 | Shortlands Convalescent ... ... | ... | ... | 99 | ... | 156 | 159 | ... | 3 | 162 |
| 10 | 7 | Woolwich and Plumstead Cottage .. | ... | ... | 73 | 30 | 513 | 413 | 11 | 92 | 516 |
| 1,701 | 1,104 | DISPENSARIES. | 5,205 | 41,703 | 15,898 | 153,236 | 96,687 | 56,613 | 34,403 | 8,639 | 99,655 |
| | | Battersea Provident ... ... ... | ... | ... | ... | 18,010 | 2,896 | 86 | 59 | 2,811 | 2,956 |
| ... | ... | Brixton, &c. ... ... ... .. | ... | ... | ... | 5,597 | 714 | 611 | 16 | 121 | 748 |
| ... | ... | Camberwell Provident ... .. | ... | 8,164 | ... | 11,538 | 2,062 | 382 | 147 | 1,413 | 1,942 |
| ... | ... | Clapham ... ... ... ... | ... | 1,880 | ... | 1,494 | 384 | 297 | ... | 112 | 409 |
| ... | ... | Deptford Medical Mission ... ... | ... | .. | ... | 3,984 | 464 | 394 | 42 | 93 | 529 |
| ... | ... | East Dulwich Provident ... ... | ... | ... | ... | 2,022 | 649 | 88 | 1 | 567 | 656 |
| ... | ... | Forest Hill ... ... ... ... | ... | ... | ... | 2,269 | 734 | 224 | 4 | 472 | 700 |
| ... | ... | Gipsy Hill, &c. ... ... ... ... | ... | ... | .. | 523 | 98 | 46 | ... | 51 | 97 |
| ... | ... | Royal South London ... ... ... | ... | 5,850 | ... | 4,947 | 622 | 566 | 134 | ... | 700 |
| ... | ... | South Lambeth, &c. ... ... ... | ... | 2,805 | ... | 2,223 | 549 | 280 | 15 | 175 | 470 |
| ... | ... | Walworth Provident ... ... ... | ... | ... | ... | 719 | 226 | 11 | 35 | 104 | 150 |
| ... | ... | Wandsworth Common... ... ... | ... | ... | ... | 780 | 192 | 46 | 147 | ... | 193 |
| ... | ... | Woolwich, &c., Provident ... ... | ... | ... | ... | 580 | 204 | 11 | 13 | 99 | 123 |
| ... | ... | Institutions not now participating ... | ... | 3,396 | ... | ... | ... | ... | ... | ... | ... |
| 1,701 | 1,104 | | 5,205 | 63,798 | 15,898 | 207,922 | 106,481 | 59,655 | 35,016 | 14,657 | 109,328 |

### CITY AND EAST CENTRAL DISTRICT.

Comprising the City, St. Luke's, Shoreditch, Finsbury, and Clerkenwell.

| No. of Beds. | No. of Beds Daily Occupied. | HOSPITALS. | 1871-1873. In-patints. | 1871-1873. Out-patients. | 1894-1896. In-patints. | 1894-1896. Out-patients. | Total Expenditure. | Income. Charitable. | Income. Proprietary. | Income. Patnts' pymnts. | Total Income. |
|---|---|---|---|---|---|---|---|---|---|---|---|
| | | | | | | | £ | £ | £ | £ | £ |
| 114 | 70 | Metropolitan ... ... ... ... | 330 | 37,012 | 1,013 | 26,768 | 8,960 | 5,944 | 460 | 612 | 7,016 |
| 170 | 139 | Royal Free ... ... ... ... | 1,406 | 46,018 | 2,070 | 36,925 | 11,225 | 5,263 | 1,047 | ... | 6,310 |
| 80 | 54 | Royal, for Diseases of the Chest ... | 157 | 4,943 | 682 | 6,583 | 7,459 | 7,152 | 153 | ... | 7,305 |
| 57 | 47 | North-Eastern, for Children ... ... | 145 | 11,431 | 705 | 15,272 | 5,572 | 6,472 | 565 | 674 | 7,711 |
| 36 | 21 | City of London Lying-In ... ... | 428 | 390 | 490 | 1,585 | 3,716 | 604 | 3,433 | ... | 4,037 |
| 45 | 18 | St. Mark's, for Fistula ... ... | 647 | 3,855 | 237 | 841 | 3,703 | 1,096 | 702 | ... | 1,798 |
| 100 | 86 | Royal London Ophthalmic ... .. | 1,200 | 20,000 | 2,006 | 26,074 | 7,246 | 2,778 | 1,017 | ... | 3,795 |
| 45 | 30 | City Orthopædic ... ... ... | ... | ... | 199 | 2,866 | 1,488 | 1,431 | 33 | ... | 1,464 |
| 17 | 11 | Central London Throat and Ear ... | ... | ... | 302 | 7,020 | 2,204 | 504 | 82 | 1,312 | 1,898 |
| 664 | 476 | DISPENSARIES. | 4,313 | 123,649 | 7,704 | 123,934 | 51,573 | 31,244 | 7,422 | 2,598 | 41,334 |
| | | Royal Maternity Charity ... ... | ... | 3,379 | | | | | | | |
| ... | ... | City ... ... ... ... ... ... | ... | 13,325 | ... | 4,843 | 1,343 | 1,088 | 184 | ... | 1,272 |
| ... | ... | City of London and East London ... | ... | 3,870 | ... | 14,999 | 1,276 | 124 | 43 | 1,334 | 1,501 |
| ... | ... | Farringdon General ... ... ... | ... | 6,088 | ... | 4,576 | 678 | 544 | ... | 260 | 804 |
| ... | ... | Finsbury... ... ... ... ... | ... | 8,265 | ... | 11,488 | 930 | 611 | 144 | 277 | 1,032 |
| ... | ... | Metropolitan ... ... ... ... | ... | 2,700 | ... | 5,978 | 1,048 | 395 | 131 | 303 | 829 |
| ... | ... | Royal General ... ... ... ... | ... | 2,458 | ... | 3,818 | 863 | 362 | 357 | 99 | 818 |
| 664 | 476 | | 4,313 | 163,734 | 7,704 | 169,636 | 57,711 | 34,368 | 8,351 | 4,871 | 47,590 |

### ST. MARYLEBONE AND WEST CENTRAL DISTRICT.

Comprising St. Marylebone, St. John's Wood, Bloomsbury, Holborn, &c.

| No. of Beds. | No. of Beds Daily Occupied. | HOSPITALS. | 1871-1873. | | 1894-1896. | | Total Expenditure. | Income. | | | Total Income. |
|---|---|---|---|---|---|---|---|---|---|---|---|
| | | | In-patints. | Out-patients. | In-patints. | Out-patients. | | Charitable. | Proprietary. | Patnts' pymnts. | |
| | | | | | | | £ | £ | £ | £ | £ |
| 70 | 50 | French ... ... ... ... ... ... | 154 | 3,046 | 879 | 4,921 | 4,070 | 3,185 | 62 | ... | 3,247 |
| 20 | 12 | Italian ... ... ... ... ... ... | ... | ... | 253 | 5,158 | 817 | 835 | 91 | ... | 926 |
| 100 | 77 | London Homœopathic ... ... ... | 484 | 21,097 | 1,031 | 14,514 | 8,376 | 3,064 | 2,546 | 598 | 6,208 |
| 51 | 43 | SS. John and Elizabeth ... ... | 103 | ... | 96 | ... | 1,660 | 694 | 890 | ... | 1,584 |
| 321 | 286 | The Middlesex ... ... ... ... | 2,016 | 20,692 | 3,598 | 44,484 | 30,483 | 10,759 | 9,296 | ... | 20,055 |
| 68 | 57 | Alexandra for Children ... ... | 66 | ... | 174 | 258 | 2,682 | 2,170 | 150 | 501 | 2,821 |
| 30 | 27 | Hospital for Incurable Children ... | ... | ... | 32 | .. | 1,067 | 438 | 47 | 383 | 868 |
| 238 | 163 | Hospital for Sick Children ... ... | 945 | 13,219 | 1,901 | 20,805 | 14,442 | 6,227 | 3,928 | ... | 10,155 |
| 18 | 10 | British Lying-In ... ... ... | 177 | 570 | 202 | 333 | 1,483 | 480 | 1,367 | ... | 1,847 |
| 55 | 44 | Queen Charlotte's Lying-In ... ... | 432 | 628 | 1,151 | 1,122 | 4,332 | 3,188 | 1,431 | 13 | 4,632 |
| 42 | 37 | New Hospital for Women ... ... | 111 | 2,925 | 500 | 12,921 | 4,296 | 2,543 | 190 | 1,299 | 4,032 |
| 50 | 48 | Samaritan Free... ... ... .. | 215 | 6,492 | 578 | 9,160 | 6,598 | 3,926 | 194 | ... | 4,320 |
| 180 | 156 | National for the Paralysed, &c. ... | 329 | 2,208 | 851 | 5,385 | 13,453 | 4,357 | 1,677 | 1,924 | 7,958 |
| 25 | 19 | Hospital for Epilepsy, &c. ... ... | 81 | 282 | 89 | 755 | 1,976 | 871 | 62 | 601 | 1,534 |
| 50 | 29 | West End, for Epilepsy, &c.... ... | ... | ... | 232 | 3,956 | 2,841 | 3,019 | 60 | 495 | 3,574 |
| 13 | 7 | Central London Ophthalmic ... ... | 76 | 5,637 | 197 | 10,523 | 1,175 | 614 | 15 | 543 | 1,172 |
| 15 | 7 | Western Ophthalmic ... ... .. | 67 | 1,865 | 165 | 7,057 | 949 | 525 | 161 | ... | 686 |
| 60 | 53 | National Orthopædic ... ... ... | ... | ... | 227 | 700 | 2,278 | 927 | ... | 1,242 | 2,169 |
| 20 | 15 | Establishment for Gentlewomen ... | 105 | ... | 119 | ... | 2,331 | 1,173 | 118 | 868 | 2,159 |
| ... | ... | National Dental ... ... ... | ... | 5,990 | ... | 35,138 | 1,422 | 1,256 | ... | 249 | 1,505 |
| 1,426 | 1,140 | DISPENSARIES. | 5,361 | 84,651 | 12,275 | 177,190 | 106,731 | 50,251 | 22,285 | 8,716 | 81,252 |
| | | Bloomsbury Provident ... ... | ... | ... | ... | 503 | 246 | 24 | ... | 195 | 219 |
| ... | ... | London Medical Mission ... ... | ... | ... | ... | 2,689 | 1,157 | 888 | 79 | 148 | 1,115 |
| ... | ... | Portland Town... ... ... ... | ... | 1,571 | ... | 1,750 | 163 | 134 | 5 | 13 | 152 |
| ... | ... | Portobello Road ... ... ... | ... | ... | ... | 184 | 73 | 20 | 4 | 30 | 54 |
| ... | ... | St. John's Wood Provident ... .. | ... | 2,641 | ... | 5,115 | 694 | 246 | 32 | 331 | 609 |
| ... | ... | St. Marylebone General ... ... | ... | 4,411 | ... | 4,492 | 1,055 | 442 | 178 | 384 | 1,004 |
| ... | ... | Western General ... ... ... | ... | 5,132 | ... | 14,045 | 1,271 | 986 | 31 | 41 | 1,058 |
| ... | ... | Institutions not now participating ... | ... | 11,200 | ... | ... | ... | ... | ... | ... | ... |
| 1,426 | 1,140 | | 5,361 | 109,606 | 12,275 | 205,968 | 111,390 | 52,291 | 22,614 | 9,858 | 85,463 |

### KENSINGTON AND WEST DISTRICT.

Comprising Kensington, Paddington, Bayswater, Chelsea, Brompton, Fulham, Hammersmith, Chiswick, Brentford, Acton, Ealing, &c.

| | | | | | | | | | | | |
|---|---|---|---|---|---|---|---|---|---|---|---|
| | | | | | | | £ | £ | £ | £ | £ |
| 351 | 290 | St. George's ... ... ... ... ... | 3,509 | 15,264 | 3,873 | 26,992 | 50,137 | 10,486 | 15,609 | ... | 26,095 |
| 281 | 245 | St. Mary's ... ... ... ... ... | 1,826 | 21,037 | 3,711 | 38,367 | 20,822 | 10,244 | 2,063 | 45 | 12,352 |
| 97 | 91 | West London ... ... ... ... ... | 377 | 21,103 | 1,641 | 32,740 | 6,800 | 4,810 | 241 | ... | 5,051 |
| 321 | 224 | Hospital for Consumption ... ... | 974 | 12,089 | 1,199 | 8,106 | 30,031 | 15,101 | 8,369 | ... | 23,470 |
| 24 | 20 | Belgrave, for Children ... ... | 96 | 889 | 314 | 4,470 | 1,507 | 1,203 | 97 | ... | 1,300 |
| 50 | 49 | Cheyne, for Sick & Incurable Children | ... | ... | 69 | ... | 2,327 | 1,861 | 212 | 486 | 2,559 |
| 40 | 28 | Paddington Green, for Children ... | ... | ... | 481 | 12,524 | 2,889 | 2,495 | 119 | 232 | 2,846 |
| 120 | 100 | Victoria, for Children... ... ... | 355 | 3,350 | 1,488 | 18,906 | 7,979 | 6,493 | 342 | 415 | 7,250 |
| 74 | 55 | Chelsea, for Women ... ... ... | ... | ... | 750 | 2,595 | 5,273 | 4,349 | 202 | 725 | 5,276 |
| 105 | 83 | Cancer ... ... ... ... ... ... | 343 | 407 | 829 | 1,576 | 13,078 | 4,205 | 2,571 | ... | 6,776 |
| 135 | 95 | Female Lock ... ... ... ... | 627 | ... | 708 | ... | 3,657 | 1,180 | ... | 2,098 | 3,978 |
| 16 | 10 | Epsom and Ewell Cottage ... ... | 128 | 4,411 | 132 | ... | 924 | 567 | 7 | 200 | 774 |
| 20 | 15 | Reigate and Redhill Cottage... ... | ... | ... | 223 | ... | 846 | 721 | 64 | 109 | 894 |
| 11 | 7 | Wimbledon Cottage ... ... ... | 53 | ... | 128 | ... | 502 | 370 | 5 | 139 | 514 |
| 13 | 4 | Hounslow Cottage ... ... ... | ... | ... | 65 | 752 | 441 | 332 | 176 | 33 | 541 |
| 1,658 | 1,316 | DISPENSARIES. | 8,160 | 74,139 | 15,571 | 147,028 | 147,213 | 65,117 | 30,077 | 4,482 | 92,676 |
| | | Brompton Provident ... ... .. | ... | ... | ... | 1,752 | 513 | 144 | 71 | 264 | 479 |
| ... | ... | Chelsea, &c. ... ... ... ... | ... | 5,816 | ... | 5,657 | 672 | 559 | 181 | ... | 740 |
| ... | ... | Chelsea Provident ... ... ... | ... | ... | ... | 401 | 281 | 14 | ... | 166 | 180 |
| ... | ... | Kensal Town Provident ... ... | ... | ... | ... | 419 | 334 | 20 | ... | 282 | 302 |
| ... | ... | Kensington ... ... ... ... | ... | 5,475 | ... | 4,182 | 657 | 553 | 48 | ... | 601 |
| ... | ... | Kilburn, Maida Vale ... ... ... | ... | 2,326 | ... | 3,277 | 524 | 392 | 50 | ... | 442 |
| ... | ... | Kilburn Provident ... ... ... | ... | ... | ... | 4,299 | 1,078 | 60 | 7 | 1,011 | 1,078 |
| ... | ... | Notting Hill Provident ... ... | ... | 6,525 | ... | 232 | 173 | 58 | ... | 56 | 114 |
| ... | ... | Paddington Provident ... .. | ... | 1,385 | ... | 3,912 | 550 | 237 | 27 | 374 | 638 |
| ... | ... | Pimlico Provident ... ... ... | ... | ... | ... | 2,300 | 622 | 15 | ... | 623 | 638 |
| ... | ... | Royal Pimlico Provident ... ... | ... | 1,668 | ... | 7,237 | 922 | 372 | 39 | 541 | 952 |
| ... | ... | Westbourne Provident ... ... | ... | 449 | ... | 973 | 408 | 49 | 43 | 326 | 418 |
| ... | ... | Institutions not now participating ... | 128 | 15,805 | ... | ... | ... | ... | ... | ... | ... |
| 1,658 | 1,316 | | 8,288 | 113,588 | 15,571 | 181,669 | 153,947 | 67,590 | 30,543 | 8,125 | 106,258 |

### ISLINGTON AND NORTH-WEST DISTRICT.

Comprising Islington, Holloway, Highbury, Hampstead, Highgate, St. Pancras, Stoke Newington, Tottenham, &c.

| No. of Beds. | No. of Beds Daily Occupied. | HOSPITALS. | 1871-1873. | | 1894-1896. | | Total Expenditure. | Income. | | | Total Income. |
|---|---|---|---|---|---|---|---|---|---|---|---|
| | | | In-patnts. | Out-patients. | In-patnts. | Out-patients. | | Charitable. | Proprietary. | Patnts' pymnts. | |
| | | | | | | | £ | £ | £ | £ | £ |
| 107 | 99 | Great Northern Central ... ... | 240 | 56,145 | 1,476 | 25,394 | 9,522 | 4,893 | 826 | 414 | 6,133 |
| 30 | 20 | Hampstead Hospital ... ... ... | ... | ... | 301 | 850 | 3,161 | 2,230 | 827 | 363 | 3,420 |
| 120 | 68 | London Temperance ... ... ... | ... | ... | 1,157 | 17,392 | 10,314 | 3,925 | 2,586 | 255 | 6,766 |
| 52 | 47 | North-West London ... ... ... | ... | 3,740 | 578 | 17,800 | 4,324 | 6,669 | 145 | 49 | 6,863 |
| 210 | 188 | University College ... ... ... | 1,832 | 17,657 | 3,020 | 43,681 | 18,507 | 10,837 | 3,838 | 44 | 14,719 |
| 60 | 58 | North London Consumption ... ... | 223 | 7,935 | 441 | 3,420 | 5,874 | 5,804 | 118 | 29 | 5,951 |
| 170 | 100 | London Fever ... ... ... ... | ... | ... | 769 | ... | 21,545 | 7,486 | 2,044 | 2,384 | 11,914 |
| 28 | 16 | Invalid Asylum... ... ... ... | ... | ... | 187 | ... | 987 | 424 | 124 | 188 | 736 |
| 16 | 14 | Children's Home Hospital, Barnet ... | .. | ... | 88 | ... | 729 | 460 | 37 | 114 | 611 |
| 10 | 8 | Enfield Cottage... ... ... ... | ... | ... | 85 | ... | 542 | 438 | 32 | 19 | 489 |
| 26 | 14 | St. Saviour's Home ... ... ... | 119 | 39 | 76 | ... | 1,711 | 1,172 | 81 | 628 | 1,881 |
| 42 | 32 | Friedenheim Home ... ... ... | ... | ... | 101 | ... | 3,300 | 3,300 | 65 | 221 | 2,953 |
| 871 | 664 | DISPENSARIES. | 2,414 | 85,516 | 8,279 | 108,537 | 76,516 | 47,005 | 10,723 | 4,708 | 62,436 |
| | | Child's Hill Provident... ... ... | ... | ... | ... | 437 | 304 | 30 | 17 | 256 | 293 |
| ... | ... | Camden Provident ... ... ... | ... | ... | ... | 919 | 277 | 14 | ... | 235 | 249 |
| ... | ... | Hampstead Provident... ... ... | ... | ... | ... | 11,182 | 1,035 | 253 | 52 | 738 | 1,043 |
| ... | ... | Holloway and North Islington ... | ... | 9,713 | ... | 3,683 | 875 | 356 | 54 | 337 | 747 |
| ... | ... | Islington... ... ... ... ... | ... | 12,266 | ... | 13,435 | 843 | 267 | 19 | 562 | 848 |
| ... | ... | St. Pancras and Northern ... ... | ... | 4,862 | ... | 1,742 | 518 | 381 | 92 | 88 | 561 |
| ... | ... | Stamford Hill, &c. ... ... ... | ... | 2,902 | ... | 4,843 | 611 | 526 | 160 | ... | 686 |
| ... | ... | Institutions not now participating ... | ... | 8,022 | ... | ... | ... | ... | ... | ... | ... |
| 871 | 664 | | 2,414 | 123,281 | 8,279 | 144,778 | 80,979 | 48,832 | 11,117 | 6,914 | 66,863 |

### WESTMINSTER DISTRICT.

Comprising Westminster City and Liberties.

| No. of Beds. | No. of Beds Daily Occupied. | HOSPITALS. | 1871-1873 In-patnts. | 1871-1873 Out-patients. | 1894-1896 In-patnts. | 1894-1896 Out-patients. | Total Expenditure. | Charitable. | Proprietary. | Patnts' pymnts. | Total Income. |
|---|---|---|---|---|---|---|---|---|---|---|---|
| | | | | | | | £ | £ | £ | £ | £ |
| 175 | 149 | Charing Cross ... ... ... ... | 1,397 | 15,841 | 2,119 | 23,686 | 15,218 | 9,218 | 1,389 | 82 | 10,689 |
| 220 | 182 | King's College ... ... ... ... | 1,697 | 33,619 | 2,513 | 25,772 | 18,433 | 9,994 | 3,614 | 168 | 13,776 |
| 212 | 185 | Westminster ... ... ... ... | 1,787 | 21,847 | 2,696 | 23,364 | 14,248 | 5,693 | 3,117 | ... | 8,810 |
| 134 | 118 | Ventnor, for Consumption ... ... | 149 | 25 | 810 | ... | 12,029 | 6,608 | 1,829 | 3,395 | 11,832 |
| 17 | 8 | Grosvenor, for Women and Children | 28 | 3,865 | 92 | 2,649 | 1,527 | 1,027 | 5 | 295 | 1,327 |
| 60 | 46 | Hospital for Women ... ... ... | 344 | 3,400 | 644 | 5,158 | 6,161 | 3,724 | 264 | 434 | 4,422 |
| 25 | 21 | National, for Diseases of Heart, &c.... | 35 | 6,039 | 144 | 1,850 | 2,163 | 1,386 | 22 | 294 | 1,702 |
| 30 | 23 | Royal Westminster Ophthalmic ... | 345 | 9,146 | 544 | 10,012 | 2,386 | 1,619 | 372 | 60 | 2,051 |
| 50 | 44 | Royal Orthopædic ... ... ... | 95 | 1,500 | 153 | 706 | 2,094 | 912 | 517 | 290 | 1,719 |
| 26 | 21 | Hospital for Diseases of the Throat... | 148 | 3,285 | 518 | 8,881 | 3,803 | 973 | 170 | 2,784 | 3,927 |
| 9 | 7 | Royal Ear ... ... ... ... | ... | ... | 276 | 2,048 | 864 | 343 | 2 | 618 | 963 |
| ... | ... | Dental ... ... ... ... ... | ... | 20,000 | ... | 42,978 | 2,015 | 1,884 | 191 | 253 | 2,328 |
| 20 | 15 | Gordon, for Fistula ... ... ... | ... | ... | 234 | 885 | 1,306 | 551 | ... | 775 | 1,326 |
| 26 | 18 | St. Peter's, for Stone ... ... ... | 58 | 964 | 474 | 4,799 | 3,517 | 1,070 | 353 | 2,237 | 3,660 |
| | | British, for Diseases of the Skin ... | 9 | 4,071 | ... | ... | ... | ... | ... | ... | ... |
| 1,004 | 837 | DISPENSARIES. | 6,092 | 123,602 | 11,217 | 152,788 | 85,764 | 45,002 | 11,845 | 11,685 | 68,532 |
| ... | ... | Public ... ... ... ... ... | ... | 12,392 | ... | 3,317 | 724 | 409 | 145 | ... | 554 |
| ... | ... | St. George and St. James ... ... | ... | 6,149 | ... | 4,188 | 609 | 817 | 14 | 17 | 848 |
| ... | ... | St. George, Hanover Square ... ... | ... | 4,938 | ... | 970 | 546 | 384 | ... | 145 | 529 |
| ... | ... | Western ... ... ... ... ... | ... | 8,146 | ... | 5,508 | 1,401 | 383 | 412 | 594 | 1,389 |
| .. | ... | Westminster General ... ... ... | ... | 10,458 | ... | 6,573 | 597 | 341 | 154 | 109 | 604 |
| 1,004 | 837 | | 6,092 | 165,685 | 11,21 | 173,344 | 89.641 | 47,336 | 12,570 | 22,550 | 72,456 |

### STRATFORD AND EAST-END DISTRICT.

Comprising Bethnal Green, Tower Hamlets, West Ham, Whitechapel, Hackney, Stepney, Limehouse, Poplar, and the East.

| No. of Beds. | No. of Beds Daily Occupied. | HOSPITALS. | 1871-1873 In-patnts. | 1871-1873 Out-patients. | 1894-1896 In-patnts. | 1894-1896 Out-patients. | Total Expenditure. | Charitable. | Proprietary. | Patnts' pymnts. | Total Income. |
|---|---|---|---|---|---|---|---|---|---|---|---|
| | | | | | | | £ | £ | £ | £ | £ |
| 125 | 105 | German ... ... ... ... ... | 1,196 | 17,755 | 1,467 | 21,194 | 9,309 | 5,878 | 2,488 | 385 | 8,571 |
| 776 | 661 | London ... ... ... ... ... | 5,262 | 43,325 | 10,430 | 158,002 | 77,430 | 21,733 | 24,248 | 206 | 46,187 |
| 64 | 51 | Poplar ... ... ... ... ... | 352 | 2,810 | 876 | 17,551 | 9,037 | 8,745 | 803 | 79 | 9,627 |
| 60 | 38 | West Ham, &c.... ... ... ... | ... | 4,737 | 547 | 21,924 | 4,086 | 3,185 | 199 | ... | 3,384 |
| 164 | 110 | City of London for Dis. of the Chest | 731 | 13,460 | 848 | 16,166 | 11,953 | 7,098 | 251 | ... | 7,349 |
| 102 | 80 | East London for Children ... ... | 328 | 5,788 | 1,496 | 34,941 | 7,506 | 5,602 | 949 | ... | 6,551 |
| 92 | 42 | Mrs. Gladstone's Home ... ... | 1,317 | ... | 743 | ... | 1,053 | 453 | 377 | ... | 830 |
| 13 | 9 | East End Mother's Home ... ... | ... | ... | 233 | 274 | 1,381 | 957 | 326 | 49 | 1,332 |
| 50 | 40 | Mildmay Mission Hospital ... ... | ... | ... | 445 | 6,183 | 4,882 | 2,767 | 1,253 | 49 | 4,069 |
| 30 | 26 | Memorial Cottage, Mildmay ... ... | ... | ... | 222 | ... | 1,845 | 742 | 969 | 166 | 1,877 |
| 1,476 | 1,162 | | 9,186 | 87,875 | 17,610 | 276,235 | 128,482 | 57,160 | 32,651 | 934 | 85,957 |
| | | DISPENSARIES. | | | | | | | | | |
| ... | ... | Eastern ... ... ... ... ... | ... | 3,093 | ... | 6,173 | 672 | 317 | 296 | 70 | 683 |
| ... | ... | Hackney Provident ... ... ... | ... | ... | ... | 776 | 259 | 26 | 5 | 205 | 236 |
| ... | ... | London ... ... ... ... ... | ... | 1,350 | ... | 2,279 | 365 | 152 | 226 | | 378 |
| ... | ... | Queen Adelaide's ... ... ... | ... | 2,627 | ... | 7,502 | 488 | 382 | 210 | 43 | 635 |
| ... | ... | Tower Hamlets... ... ... ... | ... | 2,566 | ... | 4,073 | 628 | 349 | 27 | 173 | 549 |
| ... | ... | Whitechapel Provident ... ... | ... | ... | ... | 5,065 | 747 | 19 | 24 | 723 | 766 |
| 1,476 | 1,162 | | 9,186 | 97,514 | 17,610 | 302,103 | 131,641 | 58,405 | 32,651 | 2,148 | 92,204 |

### SUMMARY OF YEARLY AVERAGES FOR THREE YEARS, 1871-73.

It will be seen from the following summary that the Voluntary Hospitals and Medical Charities of London which participated in the Hospital Sunday Fund during the twelve months ending 31st December, 1873, relieved eight hundred and eighty thousand patients at a cost of £351,109. The Total Income, including Legacies, amounted to £423,234, being £72,125 in excess of the expenditure on the year's work.

| No. of Beds. | No. of Beds Daily Occupied. | Hospitals and Dispensaries. | In-patients. | Out-t. | Total Expenditure. | Income. Charitable. | Proprietary, including Legacies. | Patients' Payments. | Total Income. |
|---|---|---|---|---|---|---|---|---|---|
| | | | | | £ | £ | £ | £ | £ |
| 724 | 434 | Newington and South District ... ... | 5,205 | 63,798 | 30,594 | 22,135 | 10,330 | 2,128 | 34,593 |
| 345 | 264 | City and East Central District ... ... | 4,313 | 163,734 | 31,257 | 22,203 | 11,117 | 1,353 | 34,673 |
| 779 | 625 | Westminster District ... ... ... ... | 6,092 | 165,685 | 62,070 | 58,046 | 40,478 | 5,426 | 103,950 |
| 886 | 690 | St. Marylebone and West Central District ... | 5,361 | 109,606 | 65,562 | 44,956 | 29,426 | 2,293 | 76,675 |
| 1,160 | 912 | Kensington and West District... ... ... | 8,288 | 113,588 | 74,014 | 50,675 | 26,861 | 4,171 | 81,707 |
| 293 | 266 | Islington and North-West District ... ... | 2,414 | 123,281 | 23,697 | 21,448 | 11,020 | 1,620 | 34,088 |
| 975 | 817 | Stratford and East-end District ... ... | 9,186 | 97,514 | 63,915 | 29,376 | 27,650 | 522 | 57,548 |
| 5,162 | 4,008 | | 40,859 | 837,206 | 351,109 | 248,839 | 156,882 | 17,513 | 423,234 |

### SUMMARY OF YEARLY AVERAGES FOR THE YEARS 1894-1896.

It will be seen from the following summary that the Voluntary Hospitals and Medical Charities of London, during the twelve months ending December 31st 1896, relieved nearly One million five hundred thousand patients at a cost of £731,790. The Ordinary income only amounted to £581,162, leaving a deficiency of £150,628 on the year's work. The Legacies received in 1896 amounted to £132,376, being £44,296 less than the amount received in 1895.

| No. of Beds. | No. of Beds Daily Occupied. | Hospitals and Dispensaries. | In-Patients. | Out-Patients. | Total Expenditure. | Income. Charitable. | Proprietary. | Patients' Payments. | Total Income. |
|---|---|---|---|---|---|---|---|---|---|
| | | | | | £ | £ | £ | £ | £ |
| 1,701 | 1,104 | Newington and South District ... ... | 15,988 | 207,922 | 106,481 | 59,655 | 35,016 | 14,657 | 109,328 |
| 644 | 476 | City and East Central District ... ... | 7,704 | 169,636 | 57,711 | 34,368 | 8,351 | 4,871 | 47,590 |
| 1,004 | 837 | Westminster District ... ... ... ... | 11,217 | 173,344 | 89,641 | 47,336 | 12,570 | 12,550 | 72,456 |
| 1,426 | 1,140 | St. Marylebone and West Central District | 12,275 | 205,968 | 111,390 | 52,991 | 22,614 | 9,858 | 85,463 |
| 1,658 | 1,316 | Kensington and West District ... ... | 15,571 | 181,669 | 153,947 | 67,590 | 30,543 | 8,125 | 106,258 |
| 871 | 664 | Islington and North-West District... ... | 8,279 | 144,778 | 80,979 | 48,832 | 11,117 | 6,914 | 66,863 |
| 1,476 | 1,162 | Stratford and East-End District ... .. | 17,610 | 302,103 | 131,641 | 58,405 | 32,651 | 2,148 | 93,204 |
| 8,800 | 6,699 | | 88,554 | 1,385,420 | 731,790 | 369,177 | 252,862 | 59,123 | 581,162 |

### THE NEED AND VALUE OF PERSONAL DEVOTION.

WHEN the question is put, "What do the personal devotion and enthusiasm of hospital workers mean to the sick and the methods applied for the alleviation of sickness and suffering in London to-day?" we may truthfully reply, "It means everything."

Without the sustained devotion and enthusiasm of the workers the stupendous task set them in the maintenance of the hospitals would be an impossible one, and not all the memories of the beneficent part these institutions have played in the social history of the nation, nor the prestige of their great traditions and far-reaching usefulness, would save them from collapse.

And if the enthusiasm and devotion of the workers, the ability to be inspired rather than depressed by difficulty and discouragement, are necessary to the maintenance of the work, they are not less indispensable to its embellishment. Let us present the case as we may, the sordid details of hospital finance will never fascinate anybody; to not a few their intrusion seems revolting; something whose presence, like poverty of any sort, is to be resented.

The pleasures of perversity are enduring, and of all potent factors in philanthropy, what can compare with caprice? In the chances of life, rich institutions, like rich people, are always surest of prizes, and of all possible depositories of gifts and legacies who so likely as those who have enough and to spare?

The hospitals have been slow to turn this knowledge to account. They have displayed their indigence, and it has proved unattractive. Let them now exhibit their wealth. What stores they have! Its evidences meet you at every turn, wealth of love and sympathy, of tenderness and self-sacrifice and devotion to duty, which make a hospital ward fruitful of living lessons.

Here, at least, there is nothing of the poverty told of in the appeal, and so heartily despised. If it exist at all, it is artistically concealed. The doctors scarcely suspect it, for their costliest prescriptions are seldom questioned. The patients know nothing of it, for all their needs are supplied, while the requirements and even the luxuries of life are not denied them.

Those who would learn how much the nation owes to the hospitals must come and see for themselves what manner of work is done and what manner of workers they are who do it. The love of duty unselfishly undertaken and carried through at any cost, which makes heroes in all callings, can nowhere be more commonly found than in the wards of our hospitals; and if one would read all the story aright, one must look for it in the hearts of the sufferers.

That is what we hope the clergy who are to preach on Hospital Sunday will do. Let them catch some of the spirit of the workers; then we may hope that, as well as telling the trite tale of poverty, and depicting the miseries of the people, they may present the case in all its splendour, setting forth boldly the moral power and riches of the hospitals; for how can they be beggars who have in their keeping the treasure of a people's health, or poor to whom all the nation is debtor?

## Figures and Tables

**Figure f1:**
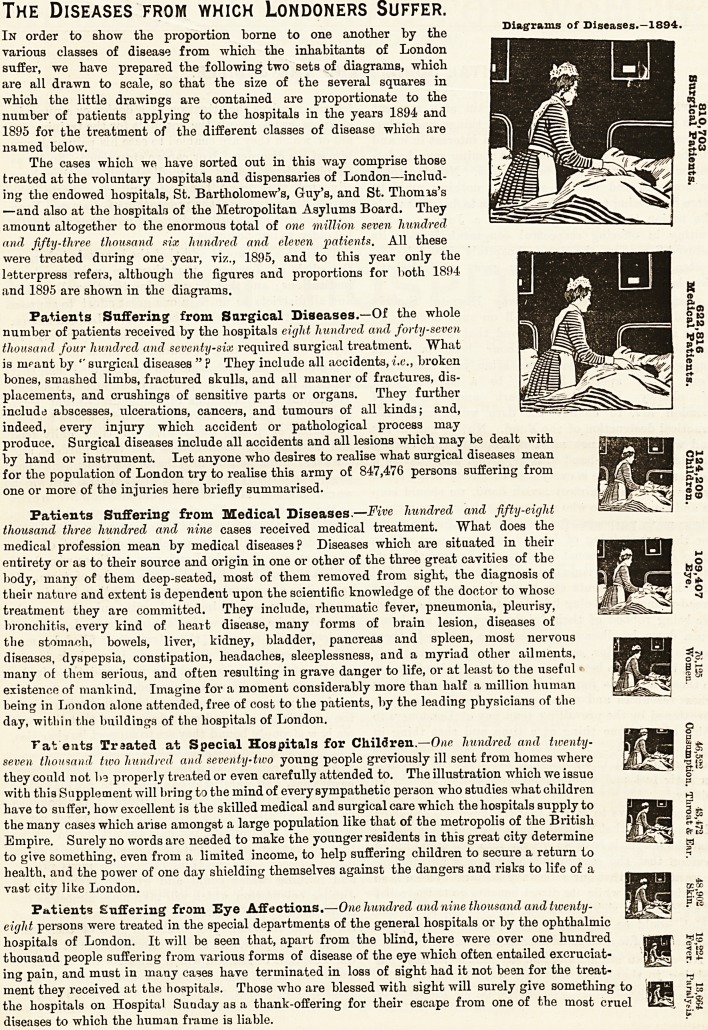


**Figure f2:**
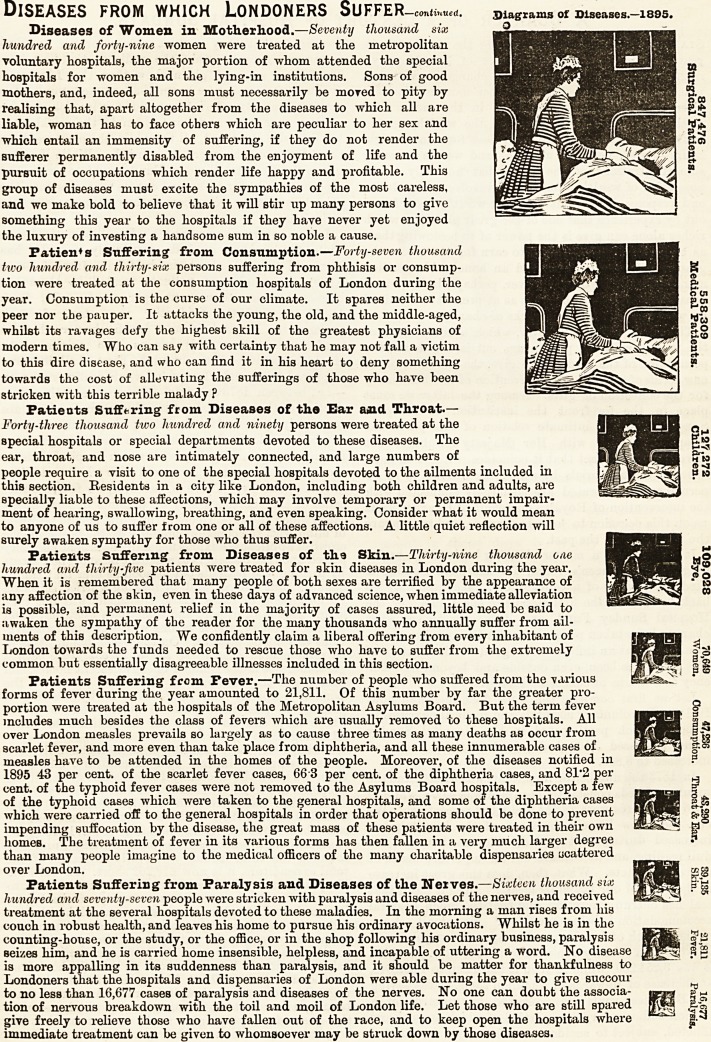


**Figure f3:**
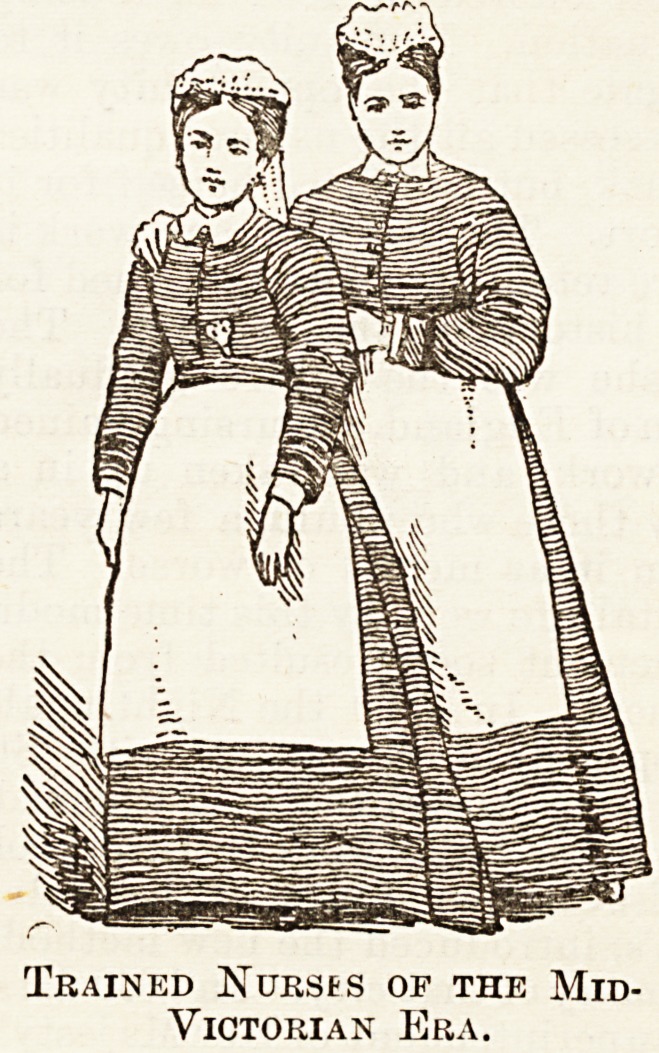


**Figure f4:**
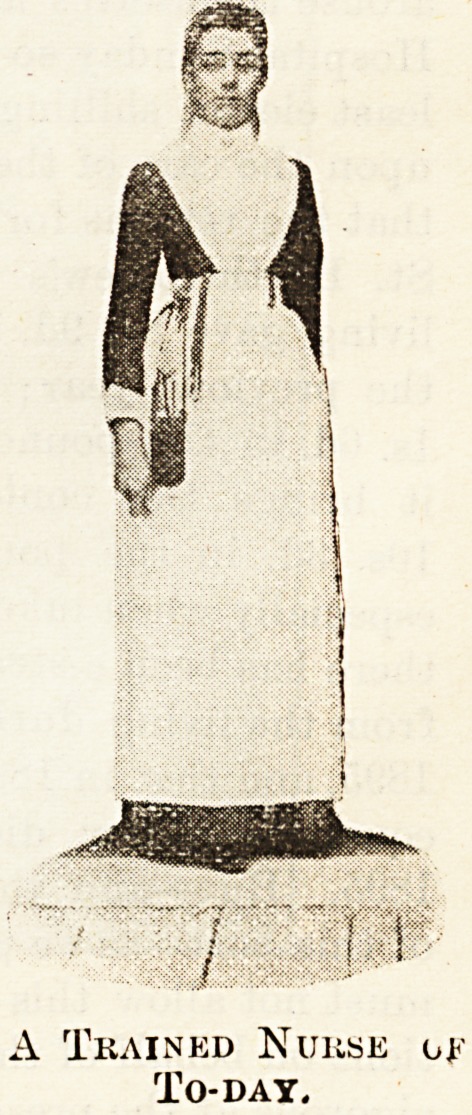


**Figure f5:**
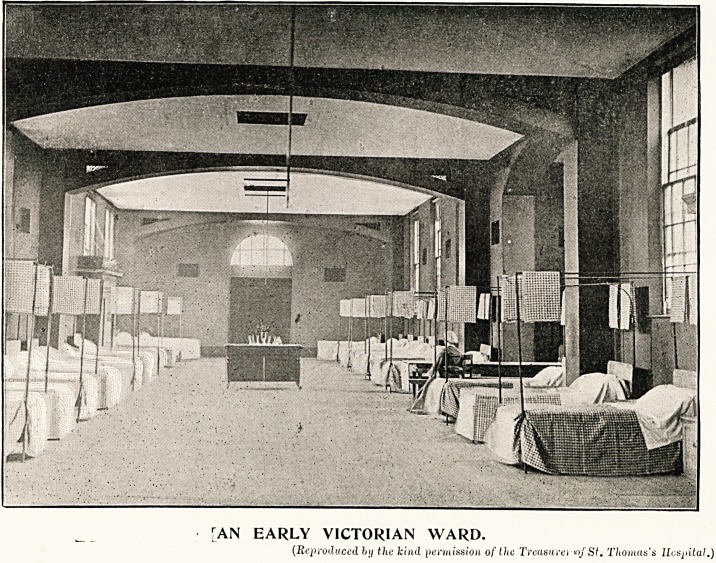


**Figure f6:**
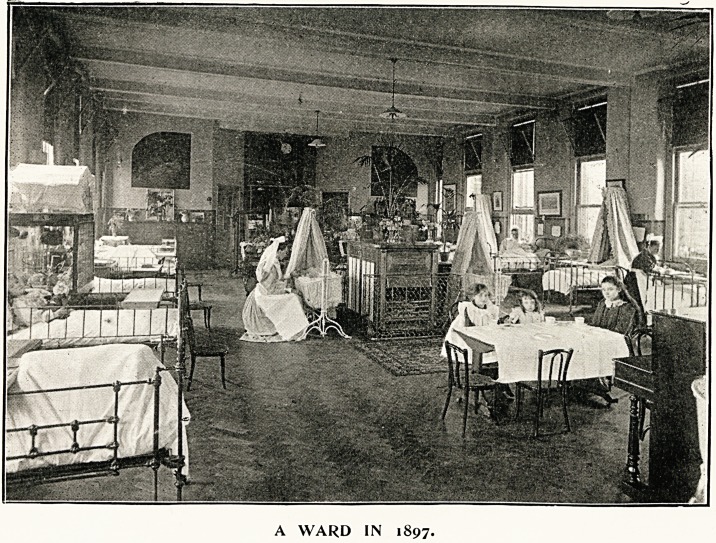


**Figure f7:**
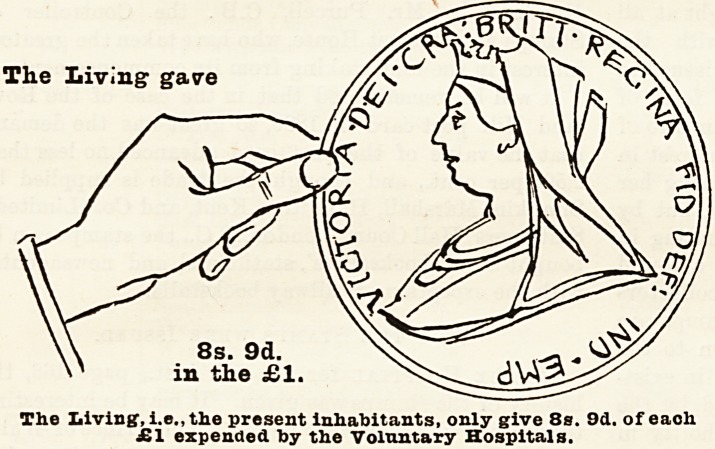


**Figure f8:**
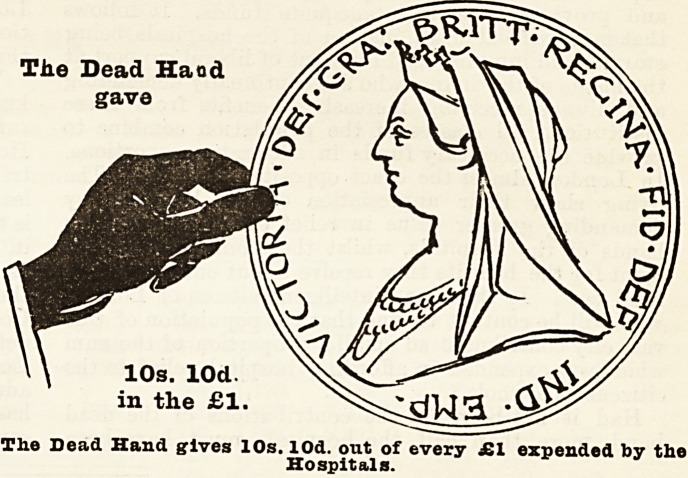


**Figure f9:**